# Systematic Changes in Gait Pursuant to Choir Participation for Caregivers and Persons With Dementia

**DOI:** 10.1093/geroni/igaa057.3036

**Published:** 2020-12-16

**Authors:** Michael Willden, Stuart MacDonald, Debra Sheets, Andre Smith

**Affiliations:** University of Victoria, Victoria, British Columbia, Canada

## Abstract

Choir interventions confer psychological benefits for persons with dementia (PwD) and their caregivers. However, less is known about whether physiological function also exhibits improvements pursuant to such social-cognitive interventions. The present study, based upon a subsample of the Voices in Motion (ViM) project, explored whether participation in an intergenerational choir results in systematic improvements in gait velocity (indexed using a GAITRite computerized walkway) for both informal caregivers (n=14; 71.4% female) and PwD (n=14; 64.3% female). Longitudinal burst data from the first of three cohorts spanning 4 assessments over 3.5 months was analysed using multilevel modeling. Whereas caregivers exhibited significant improvements (p<.05) in gait velocity, PwD showed no improvement. Ongoing analyses are exploring additional cohorts, and whether improvements in gait dynamically covary with reductions in comorbidities (e.g., neuropsychological function, caregiver burden, depressive affect). These results underscore the potential of choir for facilitating both psychosocial and physiological function for caregivers and PwD.

